# The power of randomization by sex in multilocus genetic evolution

**DOI:** 10.1186/s13062-020-00277-0

**Published:** 2020-11-23

**Authors:** Liudmyla Vasylenko, Marcus W. Feldman, Adi Livnat

**Affiliations:** 1 Department of Evolutionary and Environmental Biology and Institute of Evolution, University of Haifa, 199 Aba Khoushy Ave, Haifa, 3498838 Israel; 2 Department of Biology, Stanford University, 371 Jane Stanford Way, Stanford, 94305-5020 CA USA

**Keywords:** Random sampling, Sex and recombination, Epistasis, Randomized algorithms, Multilocus models, Interaction-based evolution

## Abstract

**Background:**

Many hypotheses have been proposed for how sexual reproduction may facilitate an increase in the population mean fitness, such as the Fisher-Muller theory, Muller’s ratchet and others. According to the recently proposed mixability theory, however, sexual recombination shifts the focus of natural selection away from favoring particular genetic combinations of high fitness towards favoring alleles that perform well across different genetic combinations. Mixability theory shows that, in finite populations, because sex essentially randomizes genetic combinations, if one allele performs better than another across the existing combinations of alleles, that allele will likely also perform better overall across a vast space of untested potential genotypes. However, this superiority has been established only for a single-locus diploid model.

**Results:**

We show that, in both haploids and diploids, the power of randomization by sex extends to the multilocus case, and becomes substantially stronger with increasing numbers of loci. In addition, we make an explicit comparison between the sexual and asexual cases, showing that sexual recombination is the cause of the randomization effect.

**Conclusions:**

That the randomization effect applies to the multilocus case and becomes stronger with increasing numbers of loci suggests that it holds under realistic conditions. One may expect, therefore, that in nature the ability of an allele to perform well in interaction with existing genetic combinations is indicative of how well it will perform in a far larger space of potential combinations that have not yet materialized and been tested. Randomization plays a similar role in a statistical test, where it allows one to draw an inference from the outcome of the test in a small sample about its expected outcome in a larger space of possibilities—i.e., to generalize. Our results are relevant to recent theories examining evolution as a learning process.

**Reviewers:**

This article was reviewed by David Ardell and Brian Golding.

## Background

Theory concerning the evolution of sex and recombination has developed along two main lines. One, modifier theory, examines the evolutionary change in the frequencies of alleles that control the rate of recombination [1–16]. The other focuses on the role of sex in evolution assuming that sex is already present (e.g., [17–20]). According to the mixability theory for the role of sex in evolution, in the presence of sexual reproduction, natural selection favors not the best specific combinations of genes; i.e., not those genotypes of highest fitness, but rather alleles that perform well in interaction with a wide variety of different genetic combinations — “mixable alleles” [21]. This theory offers an alternative view on the role of sex in evolution to the more familiar lines of work on this topic from the 20^th^ century, such as the Fisher-Muller theory [17, 18], the deterministic mutation hypothesis [20], the parasite hypothesis [19, 22] and other approaches [23–27], as well as newer lines of theory (e.g., [11, 28, 29]). Mixability theory has already had an unexpected consequence in the interdisciplinary realm: it has served as a motivation in the development of a key advance [30, 31] that contributed to the phenomenal leap of deep learning in 2012 [32, p.440] and thus to the global artificial intelligence revolution (e.g., [33]). Previous theory in evolution and in particular on the role of sexual reproduction has inspired developments in computing through the genetic algorithm work of John Holland [34], while mixability theory has inspired innovation in the science of deep learning.

Mixability theory has drawn a connection between sex and genetic evolutionary modularity [35], and has inspired work on the connection between the population genetic equations for the updates of allele frequencies in the presence of sex and natural selection with the powerful Multiplicative Weight Updates Algorithm [36], known in multiple fields under different names [37]. The mixability effect, shown initially through numerical iterations [21, 35], has also been demonstrated in a simple analytical model [38].

However, while our previous studies [21, 35, 38] have focused mostly on mixability in an infinite population context, in finite populations, an intriguing effect emerges: even though the current, finite population represents just a small sample of the space of potential genotypes [39], how well an allele performs overall in interaction with various different combinations of genetic partners in this population is indicative of how well it will perform overall in potential combinations that have not yet materialized and been tested. In other words, the interaction of natural selection and sexual recombination makes it possible for an observer to draw an inference from the success in terms of an allele’s mixability in the finite population about its potential success in an untested space of many potential genotypes [40].

Central to this effect is the idea of sex as randomization: while natural selection tests the performance of an allele as an interactant across different genetic combinations in the finite population, sexual recombination entails that the genotypes carrying that allele constitute an essentially random and thus unbiased sample of the vastly larger space of potential genotypes. Hence the outcome of natural selection in the finite population is indicative of which allele will be more mixable in a vast number of yet unseen genetic combinations [40]. Randomization plays a related role in statistical tests. In the evolutionary models described here and in statistical tests, randomization makes an outcome that is based on a small sample indicative of an outcome that would have been based on a much larger space of possibilities. In statistical testing randomization is viewed as allowing for inference-making and generalization.

This power of randomization has to date been demonstrated only in a one-locus diploid model, where interaction is between two alleles at one locus [40]. Here, we test this effect using numerical analysis of both haploid and diploid multi-locus models and demonstrate the power of randomization. In both haploid and diploid models, as the number of loci increases, selection acting on an ever smaller fraction of the space of potential genotypes suffices to infer with ever increasing accuracy which allele has the greater mixability in the space of untested potential genotypes.

Since in reality sexual species have many recombining loci—many more than can be iterated on the computer while keeping track of the space of all potential genotypes—our present results suggest that in nature alleles that are favored due to the interaction of sex and natural selection are expected to perform better as interactants in the space of yet untested genotypes.

## Results

Multilocus models allow us to examine the sexual shuffling of the genes due to recombination and/or segregation and independent assortment of chromosomes. In the haploid case, the mixability of an allele depends on its ability to interact well with a wide variety of combinations of alleles at other loci. In the diploid case, it can depend also on its ability to interact with a variety of alleles in the same locus. Here, we will consider multilocus haploid and diploid models with discrete generations, panmixia and no mutation. We will examine change across one generation only.

Consider *N* individuals, *L* loci and *n* alleles per locus. The number of possible genotypes is *n*^*L*^ in the haploid case and $\left (\frac {n(n+1)}{2}\right)^{L}$ in the diploid case. Let the fitness of genotype *G*, *w*_*G*_, be its probability of survival (we assume here for simplicity that viability, but not fertility, is genetic). Our simulations start with uniform allele frequencies, as in [40]. A starting population of *N* parents is generated by drawing at random one (haploid) or two (diploid) alleles per locus. Unless stated otherwise, the individuals can be thought of as hermaphrodites capable of selfing.

The mixability of an allele is defined as the average fitness of the genotypes carrying this allele, unweighted by their genotypic frequencies (in contrast to the marginal fitness). Formal definition of allelic mixability is given below and contrasts with fitness measures as shown in [21]. We expect that under an assumption of different mixabilities of alleles, the allele that is more mixable across all possible genotypes will increase in frequency more than the other allele, even though only a small fraction of all possible genotypes is materialized and tested by selection.

### Multilocus haploid model

Let $w_{i_{1}, i_{2}, \ldots, i_{L}}$ be the fitness of a genotype with alleles *i*_1_ at locus 1, *i*_2_ at locus 2, etc. For the *n*^*L*^ genotypes of the haploid multi-locus model with *L* loci and *n* alleles per locus, for each trial of the simulation, we randomized the fitness values $w_{i_{1}, i_{2}, \ldots, i_{L}}$ such that the two alleles of interest $\hat {i}$ and $\hat {j}$ at the first locus with mixabilities defined as $\mu _{\hat {i}} = \frac {1}{n^{L-1}} \sum \limits _{i_{2}, \ldots, i_{L}} w_{\hat {i}, i_{2}, \ldots, i_{L}}$ and $\mu _{\hat {j}} = \frac {1}{n^{L-1}} \sum \limits _{i_{2}, \ldots, i_{L}} w_{\hat {j}, i_{2}, \ldots, i_{L}}$, respectively, had a mixability ratio of $\mu _{\hat {i}} / \mu _{\hat {j}}$ equal to a pre-chosen value $d_{\hat {i}\hat {j}}$, following [40]. In this case, the mixabilities of alleles are equivalent to their marginal fitnesses because the allele frequency distribution is uniform, although allelic mixability in general is not equivalent to marginal fitness (for details, see [21]). First, fitness values $\tilde {w}$ were drawn from the normal distribution $\mathcal {N}(E,\,\sigma)$ with average *E*=0.7 and standard deviation *σ*=0.15. Thus, almost all fitness values fell in the interval [0.1]. Values not in that interval were replaced with new random numbers from the same distribution until all values were between 0 and 1. We refer to the resulting distribution as the truncated normal distribution of fitness values. Next, the fitness values of alleles $\hat {i}$ and $\hat {j}$ were adjusted as follows:

1 $$ w_{\hat{i}, i_{2}, \ldots, i_{L}} = \tilde{w}_{\hat{i}, i_{2}, \ldots, i_{L}}\sqrt{\frac{d_{\hat{i}\hat{j}} \tilde{\mu}_{\hat{j}}}{\tilde{\mu}_{\hat{i}}}}  $$

and

2 $$ w_{\hat{j}, i_{2}, \ldots, i_{L}} = \tilde{w}_{\hat{j}, i_{2}, \ldots, i_{L}}\sqrt{\frac{\tilde{\mu}_{\hat{i}}}{d_{\hat{i}\hat{j}} \tilde{\mu}_{\hat{j}}}},  $$

where $\tilde {\mu }_{\hat {i}} = \frac {1}{n^{L-1}} \sum \limits _{i_{2}, \ldots, i_{L}} \tilde {w}_{\hat {i}, i_{2}, \ldots, i_{L}}$ and $\tilde {\mu }_{\hat {j}} = \frac {1}{n^{L-1}} \sum \limits _{i_{2}, \ldots, i_{L}} \tilde {w}_{\hat {j}, i_{2}, \ldots, i_{L}}$. The adjusted values *w* have a mixability ratio $\frac {\mu _{\hat {i}}}{\mu _{\hat {j}}}=d_{\hat {i}\hat {j}}$.

Each trial of the simulation consisted of a single generation of recombination and selection. At the start of each trial, an initial population of parents was generated by drawing alleles at each of the *L* loci at random for each parent without replacement from a store of alleles at equal frequencies. Next, an offspring was generated from two random parents using the Poisson model of recombination [41, 42], according to which a crossover occurs between neighboring positions with probability *p*≤1/2, independently of crossovers at other positions. Finally, an offspring survived with probability $w_{i_{1},i_{2},\dots,i_{L}}$. This procedure was repeated until *N* surviving individuals were obtained. At the same time, the number of unique genotypes that materialized in the process—namely the number of genotypes that were tested at least once, whether they survived or not—was recorded. Finally, for each mixability ratio $d_{\hat {i}\hat {j}}$, number of alleles *n*, number of loci *L* and population size *N*, multiple independent trials were run, and the following two measurements were made: a) the across-trials average fraction of all possible genotypes that materialized and were tested by the population, *g*(*N*,*L*,*n*), and b) the fraction of trials in which, of the particular allele pair $\hat {i}$ and $\hat {j}$, the allele that was more mixable (had a higher *μ*) across all possible genotypes increased in frequency more than the allele that was less mixable across all possible genotypes, *P*(*N*,*L*,*n*) (ties in this measure were counted as “half a point” for each allele).

For clarity, we note that our results capture the fact that sex promotes the ability of alleles to perform well in the many combinations of alleles across loci that have not yet materialized, where these combinations are composed of present alleles. They do not capture the ability of alleles to perform well in interaction with alleles that have not yet been created through mutation.

**Fig. 1:**
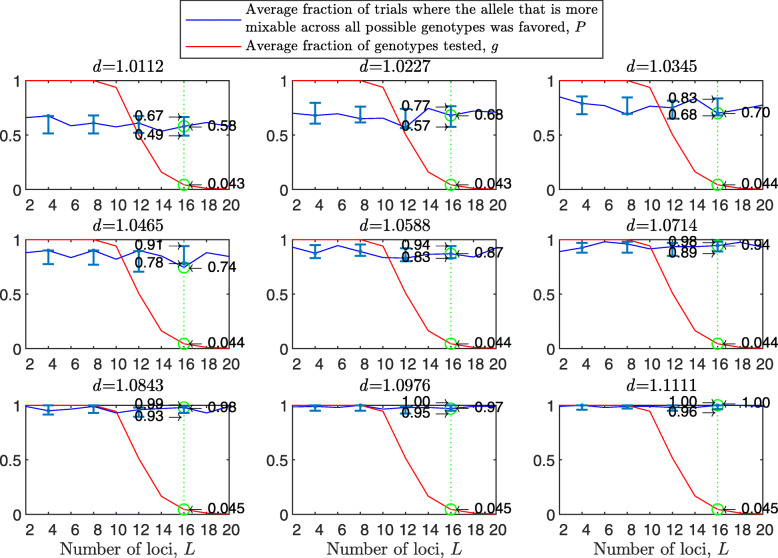
Random sampling in a multi-locus haploid model. Fitness values were drawn from the normal distribution $\mathcal {N}(0.7, \, 0.15)$. In each panel, results are shown for a population size of 2000, a varying number of loci from 2 to 20 and 2 alleles per locus. In each panel, for each number of loci, based on 100 independent trials, the red line shows *g*, the average fraction of all possible genotypes that actually materialized and were tested by the population. For each such genotype, at least one individual was born with that genotype and either survived or did not. The blue line shows *P*, the fraction of trials in which the allele that is more mixable across all possible genotypes increased in frequency more than the allele that is less mixable across all possible genotypes. Bars for the 4, 8, 12 and 16 loci cases represent a 95% confidence interval for *P* based on 80 values, each of which was obtained based on 100 independent trials

Figure 1 shows the results of such a simulation for a population size of *N*=2000 haploids, $d_{\hat {i}\hat {j}}$ values ranging from about 1.01 to 1.11, *n*=2 per locus and 100 independent trials for each parameter combination. As expected, in each panel we see that the allele that is more mixable across all possible genotypes is the one more likely to win, even though only a small fraction of all possible genotypes is actually tested. This effect increases with $d_{\hat {i}\hat {j}}$ (*P* rises across panels) and remains at the same strength when the space of potential genotypes is increased (*P* is flat within panels).

To facilitate comparisons, in all panels the green line highlights the results for the 16 locus case. On the left side of each panel, the number of loci is small, and all possible genotypes get tested. On the right side, the number of possible genotypes is large relative to the population size (2^20^≈1.05*E*6) and only a small fraction of all possible genotypes is tested. Thus, the distance between *P* and *g* increases both with $d_{\hat {i}\hat {j}}$ (owing to the increase in *P*) and with *L* (owing to the decrease in *g*). This demonstrates that sex enables random sampling in selecting for mixability: in reality, the number of loci (*L*) is large, and thus the population size becomes small relative to the number of possible genotypes, while the probability of correct evaluation remains high.

From the statistical point of view, we are comparing two distributions of fitness values (for allele $\hat {i}$ and for allele $\hat {j}$). Sex and natural selection perform the non-trivial task of distinguishing between these distributions correctly at a high probability with only a small fraction of observations drawn from these distributions and for any number of loci.

In the above, we assumed free recombination in hermaphrodites capable of selfing. To examine the case of two mating types, we divided the starting population into two separate types, “type 1” and “type 2,” and allowed mating only between types. Since the 95% confidence intervals for the two-mating-types results overlap with those of Fig. 1 almost entirely, we conclude that there is no substantial difference between hermaphrodites capable of selfing and two mating types (see Appendix Fig. 7), as in [40].

An important cause of random deviations from correct inference of mixabilities is random genetic drift due to the sampling of parents and of alleles within parents with replacement. This sampling creates random variation in the parents’ fertilities as well as in the transmission success of alleles within a parent. For a pedagogical purpose, to observe the pure effect of random sampling of genotypes by sex (which is our focus here), free of these effects of drift, one can remove drift by running the same simulations while ensuring that each haploid individual appears in exactly one mating event and produces two offspring and that each allele is transmitted exactly once. To keep the simulation simple, this scenario forces us to forgo the constant population size: instead of generating new individuals until *N* of them survive, we repeat the simulation now until *N* (even) parents have appeared in *N*/2 mating events, where each of these events creates two offspring that are complementary to each other in terms of allele transmission.

**Fig. 2:**
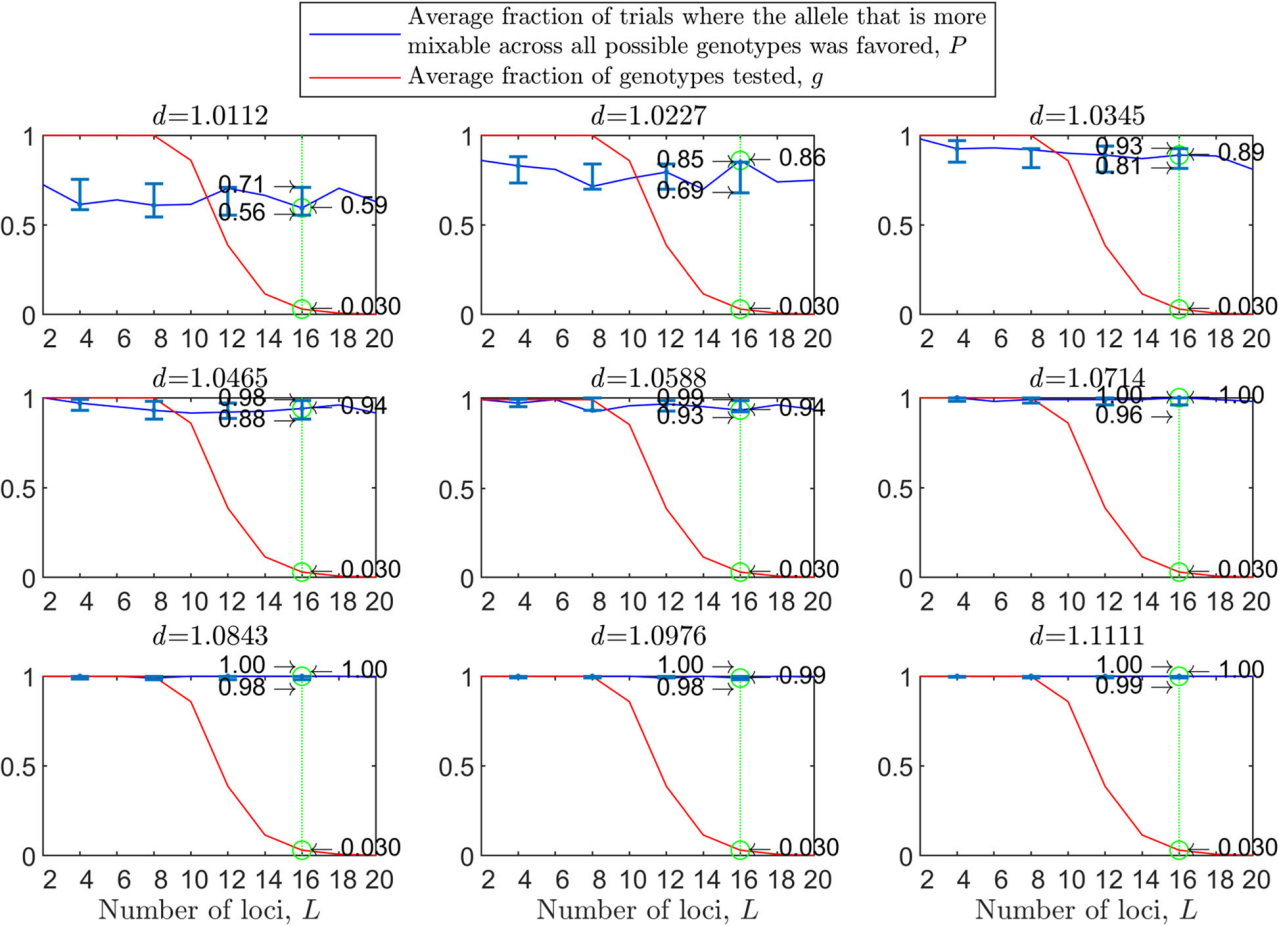
Random sampling in the multi-locus haploid model without random genetic drift. The simulation conditions are as described in Fig. 1, except that now parents are divided into two mating types, mating can occur only between type 1 and type 2 individuals, each parent participates in exactly one reproductive event that creates two offspring, and each allele in each parent is transmitted exactly once. The difference between the present figure and Fig. 1 shows the importance of drift due to the sampling of parents and of alleles with replacement

The results with random genetic drift removed (Fig. 2) are clearly stronger than those of Fig. 1. For example, with a population size of 2000, 2 alleles, 16 loci, and mixability ratio $d_{\hat {i}\hat {j}} = 1.0112$ (green line, top left panel), while in Fig. 1 selection makes the correct mixability evaluation 58% of the times by testing 4.3*%* of all possible genotypes, in Fig. 2 selection makes the correct evaluation 59% of the times by testing 3.0*%* of all possible genotypes. This evaluation reaches a rate of 98−100*%* correct with $d_{\hat {i}\hat {j}} \ge 1.08$ (all bottom panels of Fig. 2).

### All loci

Above we have tracked two alleles at one locus. How do the results change if we track two alleles at all *L* loci simultaneously?

Let us initialize the fitness matrix with random values as before from $\mathcal {N}(0.7,0.15)$ and change these values using Eqs. (1) and (2) for all loci one after the other from 1st to *L*-th in order to obtain mixability ratios between two particular alleles at each locus nearly equal to some predefined value $d_{\hat {i}\hat {j}}$ that is, for simplicity, equal across loci. Namely, let $\hat {i}_{l}$ and $\hat {j}_{l}$ be pairs of alleles at the *l*-th locus, where 1≤*l*≤*L*. Eqs. (1) and (2) are first applied to the first locus, where $\tilde {w}$ and *w* are rewritten as *w*^0^ and *w*^1^ respectively (and similarly for the *μ*s). Then the same transformation is applied to the second locus:

$$\begin{array}{*{20}l} w^{2}_{i_{1}, \hat{i}_{2}, \ldots, i_{L}}&=w^{1}_{i_{1}, \hat{i}_{2}, \ldots, i_{L}}\sqrt{\frac{d_{\hat{i}\hat{j}} \sum\limits_{i_{1}, i_{3}, \ldots, i_{L}} w^{1}_{i_{1}, \hat{j}_{2}, \ldots, i_{L}}}{\sum\limits_{i_{1}, i_{3}, \ldots, i_{L}} w^{1}_{i_{1}, \hat{i}_{2}, \ldots, i_{L}}}}\\ &= w^{1}_{i_{1}, \hat{i}_{2}, \ldots, i_{L}}\sqrt{\frac{d_{\hat{i}\hat{j}} \mu^{1}_{\hat{j}_{2}}}{\mu^{1}_{\hat{i}_{2}}}} \end{array} $$

and

$$w^{2}_{i_{1}, \hat{j}_{2}, \ldots, i_{L}}=w^{1}_{i_{1}, \hat{j}_{2}, \ldots, i_{L}}\sqrt{\frac{\mu^{1}_{\hat{i}_{2}}}{d_{\hat{i}\hat{j}} \mu^{1}_{\hat{j}_{2}}}}. $$

This procedure is repeated until finally the last locus fitness values are adjusted. The mixability ratio for alleles $\hat {i}_{l}$ and $\hat {j}_{l}$ is now precisely equal to the predefined value $d_{\hat {i}\hat {j}}$, and it has been verified by simulation that the mixability ratios for alleles at other loci are approximately equal to this value.

We now let *P* be the sum across loci of the number of trials in which, for the particular allele pair $\hat {i}_{l}$ and $\hat {j}_{l}$ at each locus, the allele that was more mixable across all possible genotypes increased in frequency more than the other allele, divided by the product of *L* and the number of trials. Results further underscore the power of the mixability effect: it is obtained for all loci simultaneously (see Appendix Fig. 8).

### Sex vs. asex in the haploid model

Previously it was shown that selection for mixability occurs in sexual and not in asexual populations [21, 35]. However, to actually observe this difference properly in a simulation is not a trivial task. That is, to draw a comparison one must start these sexual and asexual populations from the same initial conditions. Then, if mixability is measured in a multigenerational process, it takes time for the populations to diverge and begin to show a consistent difference in mixability, while at the same time the mixability measure becomes a proxy that loses power over time. Thus, the difference in mixability between sex and asex is best observed during the evolutionary transient [21]. Here and in [40] we use a different method that is based on a single generational analysis, in which starting the populations from equal beginnings poses a different but related problem: the usual way to generate an initial population would be to draw genotypes at random, but randomness is precisely the element that is supposed to be controlled for. In other words, starting at linkage equilibrium makes the asexual population, when observed through a time window of one generation, essentially a sexual one (that just lost its the ability to reproduce sexually, and hence is still at linkage equilibrium). One way of overcoming this problem is to start at perfect linkage disequilibrium—start with several clones, and in the sexual case allow only for mating between clones. In the asexual case, reproduction will copy the genotypes of the given initial clones. In the sexual case, the shuffling of the genes will produce more combinations than the initial ones, with *g* increasing with the recombination rate.

**Fig. 3:**
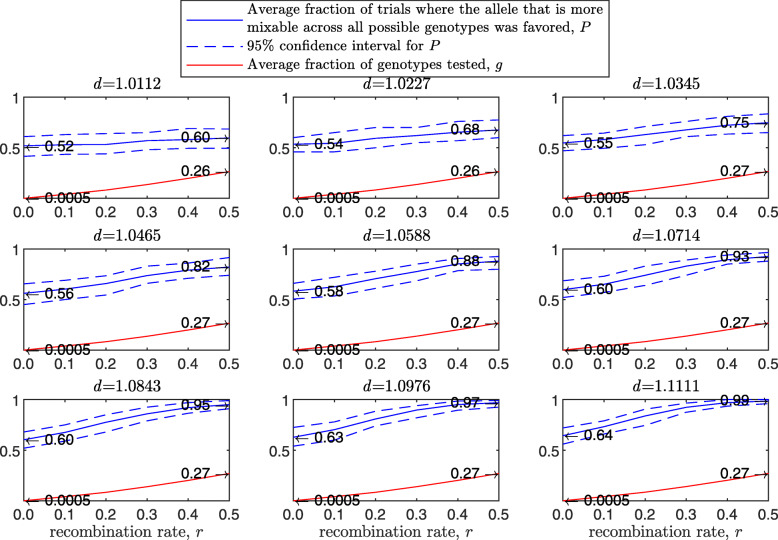
Comparison of sampling made by sex and asex in a multi-locus haploid model. Fitness values were drawn from the normal distribution $\mathcal {N}(0.7, \, 0.15)$ as described in the text. The starting population consists of two clones. In each panel, for each recombination rate from 0 (asex) to 0.5 (sex, free recombination case) on the *x*-axis, a population size of 2000, 12 loci and 2 alleles per locus, based on 100 independent trials the red line shows *g*, the blue solid line shows *P*, and the blue dashed line demarcates the 95% confidence interval of *P*, as in Fig. 1

Figure 3 demonstrates the result of such a simulation for a population size of 2000 haploids, $d_{\hat {i}\hat {j}}$ values ranging from approximately 1.01 to 1.11, number of alleles *n*=2 per locus, 12 loci and 100 independent trials for each parameter combination. The starting population consists of two clones; that is, let 0_*l*_ and 1_*l*_ be the first and second alleles, respectively, at locus *l*∈(1,…*L*). In this notation, the first clone is (0_1_,0_2_,…,0_*L*_) and the second is (1_1_,1_2_,…,1_*L*_). In the top-left panel, where $d_{\hat {i}\hat {j}}=1.0112$, the alleles are almost equally mixable, and *P* varies from 0.52 in the asexual case (no recombination; left end of panel) to 0.60 in the free recombination sexual case (right end of panel). The difference stands out in the central-left panel, where $d_{\hat {i}\hat {j}}=1.0465$ (it increases from 0.56 in the asexual case to 0.82 in the free recombination case) and reaches its maximum in the bottom-right panel, where $d_{\hat {i}\hat {j}}=1.1111$, (from 0.64 in the asexual case to 0.99 in the sexual one). Understandably, the number of tested genotypes, *g*, increases with the recombination rate.

**Fig. 4:**
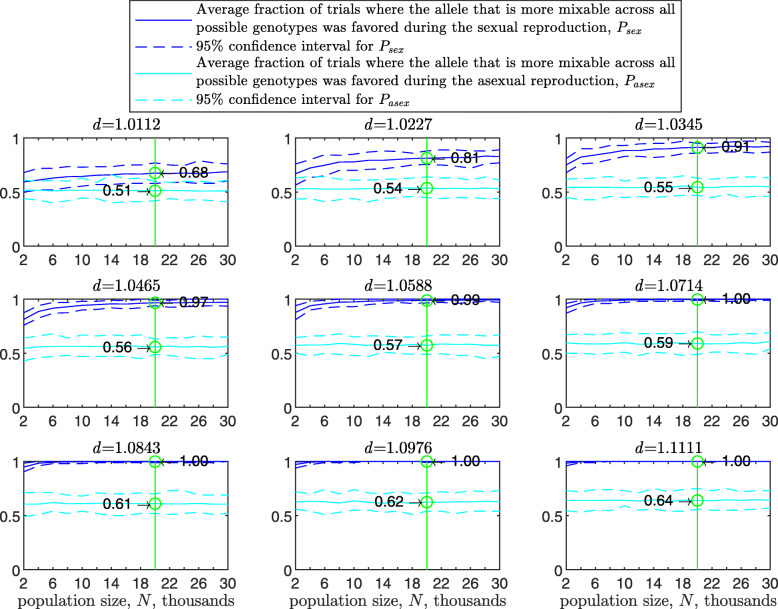
Comparison of sampling by sex and by asex in the multi-locus haploid model for different population sizes. The simulation conditions are as described in Fig. 3, except that now the population size varies on the *x*-axes and only two recombination rates values are used, *r*=0 (asex, cyan solid line; 95% C.I. cyan dashed lines) and *r*=0.5 (sex, blue solid line; 95% C.I. blue dashed lines). The probability that the more mixable allele across all possible genotypes was favored, *P*, is markedly higher in the sexual case. Furthermore, as the population size is increased, *P* increases in the sexual population but not in the asexual one. This figure shows that with increasing population size, selection for mixability becomes stronger only in the sexual population

As the population size increases, *P* increases for the sexual population but remains the same for the asexual one (Fig. 4). As the standard deviation of the fitness distributions is increased, *P* decreases much faster for the asexual than for the sexual population (Appendix Fig. 9). These results clearly demonstrate the power of randomization due to sex.

### Comparison of the simulation with theoretical probabilities in the haploid model

The probability *P* can also be examined from a statistical perspective where we are dealing with two distributions: one for the fitness values of the genotypes carrying one allele, and another for the other allele (distributions that would partly overlap in the diploid case). The question then is how well natural selection can distinguish correctly which distribution has the higher mean: in the sex case, based on comparing small random samples from these distributions, and in the asex case simulated above, based on one observation from each distribution. In the latter case, it makes the correct evaluation if the observation (clone) with higher fitness belongs to the distribution with the higher mean. Thus, the asexual probability of correct evaluation, *P*, can be directly calculated if the joint distribution of the random variables is known, a calculation which is greatly simplified when those random variables are independent. Thus, let *X* and *Y* be independent random variables with probability density functions *f*_*X*_(*x*) and *f*_*Y*_(*y*), representing the fitness value distributions of genotypes carrying allele $\hat {i}$ and allele $\hat {j}$, respectively. Since they are independent, their joint probability density function is the product of their individual probability density functions: *f*_*X*,*Y*_(*x*,*y*)=*f*_*X*_(*x*)·*f*_*Y*_(*y*), and the probability that one random variable is greater than another is

3 $$ {}P(X\!<\!Y) = \iint \limits_{x< y} f_{X,Y}(x,y) dx dy = \iint \limits_{x< y} f_{X}(x) f_{Y}(y) dx dy.  $$

In the sexual case, in contrast, averages of *N* points from each distribution are compared. Specifically, let *X*_1_,*X*_2_,…,*X*_*N*_ be independent random variables with the common density function *f*_*X*_ and *Y*_1_,*Y*_2_,…,*Y*_*N*_ be independent random variables with the common density function *f*_*Y*_. Let *E*_*X*_,*E*_*Y*_,*σ*_*X*_,*σ*_*Y*_ be the expectations and standard deviations of *X* and *Y*, respectively, $A_{N} = \frac {X_{1} + X_{2} + \cdots + X_{N}}{N}, B_{N} = \frac {Y_{1} + Y_{2} + \cdots + Y_{N}}{N}$, and *f*_*A*_ and *f*_*B*_ be the probability density functions of the normal distributions $\mathcal {N}_{A}=\mathcal {N}\left (E_{X}, \sigma _{X} / \sqrt {N}\right)$ and $\mathcal {N}_{B}=\mathcal {N}\left (E_{Y}, \sigma _{Y} / \sqrt {N}\right)$, respectively. Then, by the central limit theorem (CLT), for sufficiently large *N*, the random variable *A*_*N*_ has approximately the distribution $\mathcal {N}_{A}$ and the random variable *B*_*N*_ has approximately the distribution $\mathcal {N}_{B}$, and the probability that the average of *N* randomly selected points from one distribution is bigger than average of *N* randomly selected points from another can be calculated as follows:

4 $$ {}\begin{aligned} P(A_{N} < B_{N})&=\! \iint \limits_{a< b} f_{A,B}(a,b) \, da \, db\\ &= \iint \limits_{a< b} f_{A}(a) f_{B}(b) \, da \, db. \end{aligned}  $$

A comparison shows that, as the population size is increased (2000 and bigger), the simulated *P* comes closer to the theoretical *P* in Eq. (4) (Fig. 4 and Table 1).

**Table 1:** Comparison of theoretical and simulated probabilities, haploid case

	*P* in the sex case			*P* in the asex case	
*d*	Simulated 95% confidence interval		Theoretical value	Simulated 95% confidence interval	Theoretical value
	With genetic drift	Without genetic drift			
1.0112	(0.50,0.69)	(0.56,0.71)	0.88	(0.42,0.61)	0.5064
1.0588	(0.80,0.93)	(0.94,1.00)	1−1.7*e*^−10^	(0.51,0.66)	0.5691
1.1111	(0.96,1.00)	(0.99,1.00)	1−1.0*e*^−31^	(0.56,0.72)	0.6327

The comparison uses a population size of 2000 haploids, 12 loci and 2 alleles per locus.

### Multilocus diploid model

In our diploid model, there are no position effects; hence the fitness of a genotype with alleles (*i*,*j*) at the *l*-th locus has the same fitness value as a genotype with alleles (*j*,*i*) at that locus, yielding $\left (\frac {n(n+1)}{2}\right)^{L}$ different genotypes. For each trial of the simulation, we randomize the fitness values $w_{i_{1} j_{1}, i_{2} j_{2}, \ldots, i_{L} j_{L}}$ such that the two alleles of interest $\hat {i}$ and $\hat {j}$ from the first locus with mixabilities defined as $\mu _{\hat {i}} = \frac {1}{n\cdot \left (\frac {n(n+1)}{2}\right)^{L-1}} \times \sum \limits _{k, i_{2}, j_{2}, \ldots, i_{L}, j_{L}} w_{\hat {i}k, i_{2} j_{2}, \ldots, i_{L} j_{L}}$ and $\mu _{\hat {j}} = \frac {1}{n\cdot \left (\frac {n(n+1)}{2}\right)^{L-1}} \times \sum \limits _{k, i_{2}, j_{2}, \ldots, i_{L}, j_{L}} w_{\hat {j}k, i_{2} j_{2}, \ldots, i_{L} j_{L}}$, respectively, have a mixability ratio of $\mu _{\hat {i}} / \mu _{\hat {j}}$ almost equal to a pre-chosen value $d_{\hat {i}\hat {j}}$. Due to computational restrictions, the simulation was performed for *n*=2 alleles per locus. As in the haploid model, fitness values $\tilde {w}$ were first drawn from $\mathcal {N}(0.7, \, 0.15)$ and then truncated. Then, the fitness values of alleles $\hat {i}$ and $\hat {j}$ were adjusted as follows:

5 $$ \begin{aligned} w_{\hat{i}k, i_{2} j_{2}, \ldots, i_{L} j_{L}} = \tilde{w}_{\hat{i}k, i_{2} j_{2}, \ldots, i_{L} j_{L}} \sqrt{\frac{\left(2d_{\hat{i}\hat{j}}-1\right)\sum\limits_{l\neq\hat{i}; i_{2}, j_{2}, \ldots, i_{L}, j_{L}} \tilde{w}_{\hat{j}l, i_{2} j_{2}, \ldots, i_{L} j_{L}}}{\sum\limits_{l\neq\hat{j}; i_{2}, j_{2}, \ldots, i_{L}, j_{L}} \tilde{w}_{\hat{i}l, i_{2} j_{2}, \ldots, i_{L} j_{L}}}} \end{aligned}  $$

and

6 $$ \begin{aligned} w_{\hat{j}k, i_{2} j_{2}, \ldots, i_{L} j_{L}} = \tilde{w}_{\hat{j}k, i_{2} j_{2}, \ldots, i_{L} j_{L}} \sqrt{\frac{\sum\limits_{l\neq\hat{j}; i_{2}, j_{2}, \ldots, i_{L}, j_{L}} \tilde{w}_{\hat{i}l, i_{2} j_{2}, \ldots, i_{L} j_{L}}}{\left(2d_{\hat{i}\hat{j}}-1\right)\sum\limits_{l\neq\hat{i}; i_{2}, j_{2}, \ldots, i_{L}, j_{L}} \tilde{w}_{\hat{j}l, i_{2} j_{2}, \ldots, i_{L} j_{L}}}} \end{aligned}  $$

(see the Appendix “Obtaining mixability ratios in the diploid case” section).

**Fig. 5:**
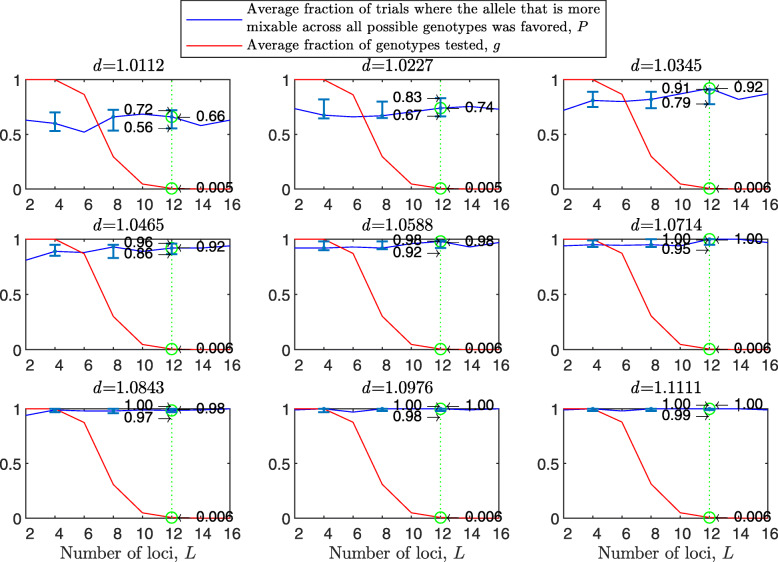
Random sampling in a multi-locus diploid model. The results were produced and presented in a manner analogous to Fig. 1, the difference being that this model is diploid and number of loci ranges from 2 to 16. Results are much stronger than in the haploid case

Figure 5 shows that the diploid case results are stronger than the haploid ones. For example, the 95% confidence interval for *P* over all loci tested here is included in 0.56−0.72 for the diploid vs. 0.52−0.68 for the haploid for $d_{\hat {i}\hat {j}} = 1.0112$; 0.92−0.98 diploid vs. 0.84−0.95 haploid for $d_{\hat {i}\hat {j}} = 1.0588$; and 0.99−1 vs. 0.96−1 for $d_{\hat {i}\hat {j}} = 1.1111$. *P* increases with *d* and varies little across panels. Results for two mating types are similar to Fig. 5 (see Appendix Fig. 10), and much stronger with random genetic drift removed (Appendix Fig. 11). However, the reason that the diploid results are stronger appears to be that in the diploid model the fitness difference between the homozygotes at a given locus is bigger than that the fitness difference between two alleles at a given haploid locus for the same mixability ratio because of the existence of the heterozygote genotype in the former, and only the homozygotes $\hat {i}\hat {i}$ and $\hat {j}\hat {j}$ contribute to *P* (the *P* that relates to $\hat {i}$ and $\hat {j}$ at the given locus). This effect decreases with the number of alleles (Appendix Fig. 12).

### Sex vs. asex in the diploid model

Given the two alleles 0_*l*_ and 1_*l*_ at each locus 1≤*l*≤*L* and the homozygous clones (0_1_0_1_;0_2_0_2_;…;0_*L*_0_*L*_) and (1_1_1_1_;1_2_1_2_;…;1_*L*_1_*L*_), any sexual mating between clones will produce the same *F*1 genotype (0_1_1_1_;0_2_1_2_;…;0_*L*_1_*L*_). Therefore, to compare sex and asex in the diploid case, we simulated two generations and compared the starting population with the second generation’s population.

**Fig. 6:**
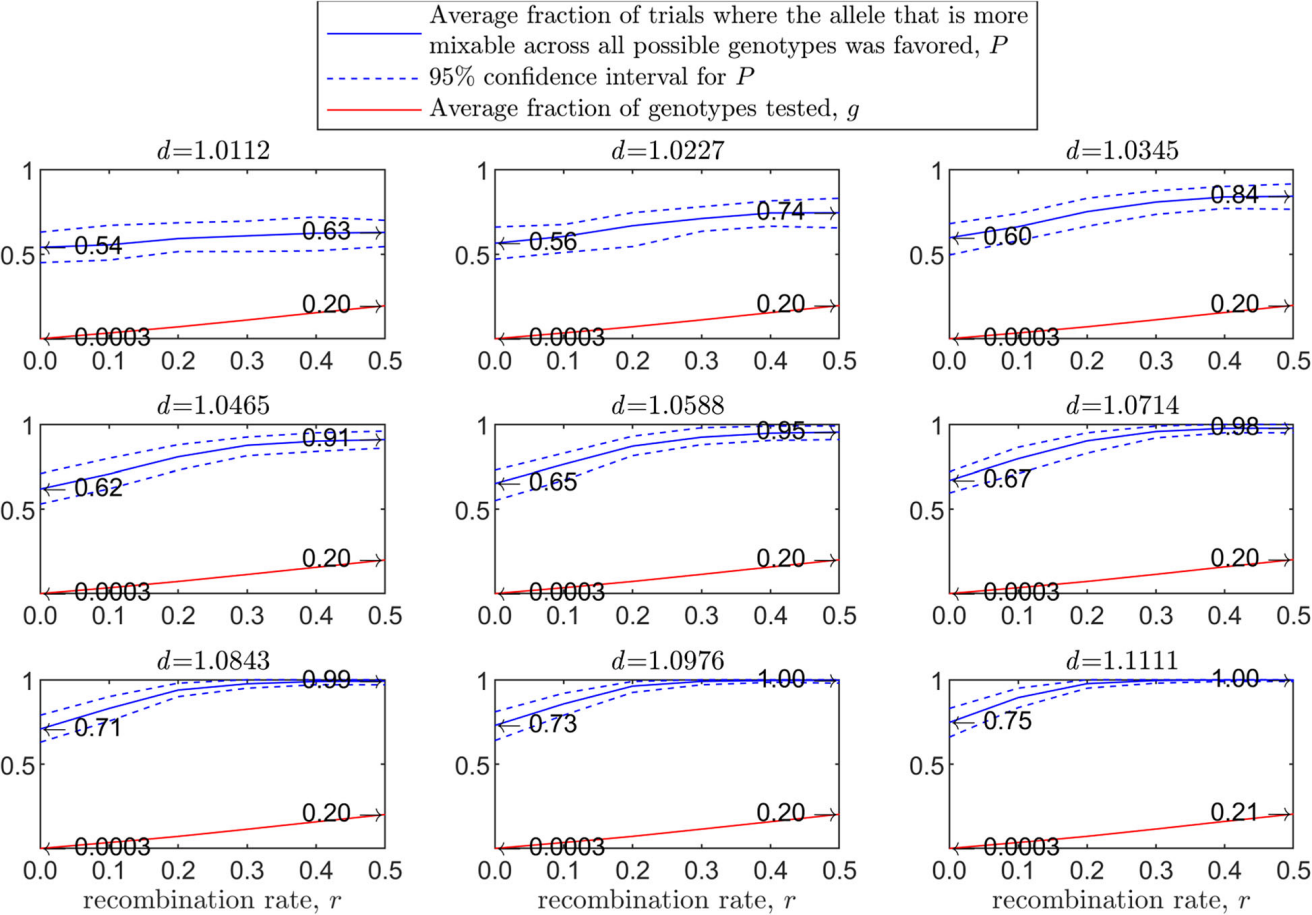
Comparison of sampling by sex and by asex in a multi-locus diploid model. Fitness values were drawn from the normal distribution $\mathcal {N}(0.7, \, 0.15)$. In each panel, results are shown for a population size of 2000, 8 loci and 2 alleles per locus. The simulation conditions are as described in Fig. 3, the difference being that this model is diploid

Results of this simulation for a population size of 2000 diploid individuals, $d_{\hat {i}\hat {j}}$ values ranging from 1.01 to 1.11, number of alleles *n*=2 per locus, 8 loci and 100 independent trials for each parameter combination are presented in Fig. 6. In comparison to Fig. 3, *P* is larger for both the asex and free recombination cases across panels. The difference in *P* between sex and asex in Fig. 6 increases faster with *d* than in Fig. 3 (first 5 panels) and then decreases due to a ceiling effect. As in the haploid case, *P* increases with the population size for the sexual population but remains the same for the asexual one (Appendix Fig. 13). Again as in the haploid case, as the standard deviation of the fitness distributions is increased, *P* decreases much faster for the asexual than for the sexual population (Appendix Fig. 14).

### Comparison of the simulation with theoretical probabilities for the diploid case

The probability *P* in the asexual case can be calculated theoretically, if the joint distribution of random variables *X* and *Y* from expression (3) is known. However, the fitness value distributions for two alleles of interest overlap in the diploid case, hence they are not independent and the simplification in the second equation of (3) can not be used. Consider the distributions of fitness values for homozygotes at the first locus for genotypes with two alleles per locus. Let $\tilde {X}$ and $\tilde {Y}$ be random variables from the distributions $f_{\tilde {X}}$ and $f_{\tilde {Y}}$ of genotypes with $\hat {i}\hat {i}$ and $\hat {j}\hat {j}$ respectively at the first locus, which are independent. Now,

7 $$ {}\begin{aligned} P\left(\tilde{X}\! <\! \tilde{Y}\right) = \iint \limits_{x< y} f_{\tilde{X},\tilde{Y}}(x,y) dx dy = \iint \limits_{x< y} f_{\tilde{X}}(x) f_{\tilde{Y}}(y) dx dy \end{aligned}  $$

(see the Appendix “Derivation of expression (7)” section). The ratio between the expectations of $\tilde {X}$ and $\tilde {Y}$ is equal to $2d_{\hat {i}\hat {j}}-1$ (see Eqs. (5), (6)). It is greater than that between the expectations of *X* and *Y*, which is equal to $d_{\hat {i}\hat {j}}$, because of the heterozygous genotype (see the Appendix “Obtaining mixability ratios in the diploid case” section for details). Therefore, the difference between sex and asex in the diploid case is greater than in the haploid case. Appendix Fig. 14 shows that it increases with *σ*. By the CLT, the mean of *N* points from one distribution has the distribution $\mathcal {N}(E,\sigma / \sqrt {N})$ for large enough *N*. Therefore, equation (4) can be used here. In Table 2 it is shown that the simulated *P* value is close to the theoretical one.

**Table 2:** Comparison of theoretical and simulated probabilities, diploid case

	*P* in the sex case			*P* in the asex case	
*d*	Simulated 95% confidence interval		Theoretical value	Simulated 95% confidence interval	Theoretical value
	With genetic drift	Without genetic drift			
1.0112	(0.55,0.70)	(0.62,0.78)	0.9901	(0.45,0.63)	0.5236
1.0588	(0.91,0.99)	(0.96,1.00)	1−2.2*e*^−35^	(0.55,0.73)	0.6393
1.1111	(0.99,1.00)	(1.00,1.00)	1−5.4*e*^−107^	(0.66,0.83)	0.6918

The comparison uses a population size of 2000 diploids, 8 loci and 2 alleles per locus.

## Discussion

In both haploid and diploid cases, we find that sex has the power of randomization: by essentially randomizing genetic combinations, the allele that is favored by natural selection in its interactions with the existing genetic combinations in a current, finite population is also likely to perform better overall across the much larger space of untested, potential genotypes. The results extend our previous studies [40] to the multilocus case. Indeed, increasing the number of loci substantially strengthens the effect: as the number of loci increases, an ever smaller fraction of the space of potential genotypes needs to be tested in order for selection to favor the allele that will most likely also be mixable across the many untested potential genotypes, with ever increasing accuracy. In addition, we demonstrate the power of randomization due to sex by directly comparing sex and asex, showing that selection favors the more mixable alleles substantially more in the sexual population, more so for larger populations and intermediate fitness variance. For sufficiently small *σ*, even one randomly selected point is sufficient to distinguish two distributions, i.e. the accuracy in the asexual case is high and is therefore close to the sexual one. For sufficiently large *σ*, the distributions are very close, and even many randomly selected points are not enough to distinguish these distributions, i.e. the accuracy in the sexual case is low, close to the asexual one.

To better understand the idea of sex as randomization, it is useful to contrast it with previous theories, such as the Fisher-Muller theory of the benefit of sex [17, 18]. In the latter, sex allows for parallel as opposed to serial accumulation of beneficial mutations: beneficial mutations at different loci that originated in different individuals can be combined into one individual, whereas in an asexual population, such mutations must occur serially in the same clone in order to accumulate under natural selection [17, 18, 43, 44]. However, that theory assumes a priori that a beneficial allele is favored over the wild-type no matter what genetic combination it is in—it is “beneficial” in the sense that it has a value of its own, independent of alleles at other loci, and once it arises, it spreads to fixation because of this rather independent effect [17, 18, 43, 44]. In that framework, there is no need for selection to explore the value of an allele over the generations, because the allele is understood to be beneficial from the start, independently of the genetic context. In the present analysis our focus is on the mixability of alleles as the measure of interest, rather than the population mean fitness, while allowing for genetic interactions.

Another surprising implication of the results is as follows. Both in the case where genes do not interact, and in the case where they do interact but in a random fashion (where the fitness of a genotype is a random function of its constituent alleles—the most complex function in the Kolmogorov complexity sense [45]), there is no information to be gained on the mixability of alleles by random sampling of potential genotypes and their fitnesses [40]. Therefore, if the power of randomization by sex is important in nature, then genetic interactions must be common and structured—they must be not overly complex [40]. This implication further underscores the difference between the idea of sex as harnessing the power of randomness [40] and previous theories on the role of sexual reproduction in evolution. For example, the deterministic mutation hypothesis requires a more restrictive form of genetic interaction [20].

In evolutionary theory, randomness has been seen as a force that leads directly to new genetic information: Random mutation represents random change in the information that is stored in the genome, a change that may sometimes contribute to a beneficial phenotypic change. A recombination event can result in a beneficial change to the extent that it can create a lasting beneficial combination of alleles, for example as in the Shifting-Balance Theory [46, 47]. Here we show a very different way by which randomness can be important in evolution: it can be harnessed in an effective way, not as a force that leads to new genetic information directly, but as an element of a larger system. In our case, it makes natural selection in a finite population act in a manner indicative of the ability of alleles to perform well as interactants in the space of untested potential genotypes [40]. Indeed, the fact that randomization can be harnessed very effectively as a part of a bigger system is well known. In the experimental sciences, it is used for random sampling or random assignment to conditions. In computer science, many algorithms have been created that use randomization in an effective way, from testing whether an algebraic identity is correct, to encrypting messages, to testing software, to sorting large files, and more [48, 49]. However, this well-known effect of randomization has not previously been proposed as a possibly important element in the process of evolution.

A better understanding of sexual reproduction may be relevant not only to population genetics but also to computer science. It is well known that a simple hypothesis that explains many different facts is a good hypothesis [45]—it does not suffer from over-fitting and is more likely to be correct. Mixable alleles are alleles that work well in many different genetic contexts, and can be viewed as simple modules [21, 30, 31, 35]. In this light, sex may be seen as a phenomenon that decomposes the genome into recombining loci where a mixable allele represents a good, simple “hypothesis” about what genetic information at a given locus will work well in interaction with the genetic information at other loci.

Viewing mixability as nature’s way of simplifying interactions between genes, Hinton and colleagues designed an analogous method for the training of deep learning neural networks [30, 31] called “dropout,” where 50% of the units in the network are chosen at random and temporarily dropped out of the network at each instance of training. This prevents the appearance over time of units that work well only in the context of specific other units, and favors instead the appearance of units that perform well across different contexts, as in the mixability effect of sex on alleles [30, 31]. This serves as a form of simplification of the interactions between units and as a means of preventing over-fitting while creating robust units [30, 31]. The resulting algorithm was described as one of four breakthroughs that allowed for the comeback of artificial neural networks through deep learning in 2012 [32, p. 440], which in turn has been a key part of the recent global artificial intelligence revolution (e.g., [33]).

Relatedly, because the interaction of sex and natural selection acts in a manner that is indicative of the performance of alleles in future genetic combinations, and because inference-making is a central aspect of learning, our finding naturally connects with recent work proposing that evolution can be viewed as a learning process (e.g., [36, 50–54]). Both the theory of Interaction-based Evolution [51, 54] and Evolutionary Connectionism [52, 53] recognize the importance of simplification in learning processes but approach simplification in biological evolution in different ways. According to Interaction-based Evolution (IBE) theory, simplification can be implemented directly by mechanisms of genetic change [54]. Therefore, parsimony can serve as a central force in evolution, and natural selection on the one hand and genetic mechanisms of simplification on the other can interact and allow for evolution by the combination of parsimony and fit [54]. In contrast, the evolutionary connectionist approach took methods of simplification known in machine-learning and introduced them into evolutionary simulations of gene-regulatory networks (GRNs) to some beneficial effect [55] but did not ground this simplification biologically in a way that would explore its relevance and importance to biological evolution.

The present results exemplify this difference. One way by which simplification is forced into the simulations of the evolutionary connectionist approach is by introducing Gaussian noise to the “target phenotype” at each generation [55]. Interestingly, however, the authors of ref. [55] connect their approach to dropout: “Masking spurious details in the training set by adding noise to the training samples during the training phase is a general method to combat the problem of over-fitting in learning systems. This technique is known as ‘training with noise’ or ‘jittering’... and is closely related to the use of intrinsic noise in deep neural networks; a technique known as ‘dropout’ ” [55, p.9]. However, note that *a*) dropout was motivated by mixability theory [31]; *b*) from the point of view of mixability, sexual recombination was seen as nature’s way of simplifying interactions between loci [21, 30, 31, 35]; and *c*) sexual recombination is a quintessential example of a mechanism of genetic change. Thus, the quote from ref. [55] actually returns us to the position of IBE [51, 54], which focuses on the centrality of mechanisms of genetic change. That is, we have demonstrated that randomization is directly inserted into the evolutionary process in nature by sexual recombination itself. Sexual recombination decomposes the genome into simple units or modules [21, 30, 31, 35], where an allele will be favored at a focal locus if it is a better, simple, generalizable hypothesis about what information will work well with other pieces of information at other loci. Thus, simplification in evolution can be implemented by mechanisms of genetic change. Indeed, both in the cases of evolution and statistical tests, randomization allows for an outcome based on a small sample to be indicative of the outcome that would emerge from a far larger space of possibilities. In the case of evolution, it allows selection to act as a signal of the mixability of an allele in future genetic combinations. In the case of statistical tests, randomization allows for inference and generalization, which are key aspects of learning processes.

## Conclusions

The theoretical study of the role of sex in evolution traditionally focused on the question of how sex might facilitate the increase in population mean fitness. However, this focus is insufficient to explain the evolution of this complex adaptation because the mean fitness does not necessarily capture complex biological structure. Mixability theory takes an alternative approach that focuses on the ability of alleles to perform well as interactants across a wide variety of different genetic combinations and how the sexual shuffling of the genes affects this performance. We found that in both haploid and diploid multilocus systems, alleles that performs better across existing genetic combinations are also the ones most likely to perform better across the much larger space of untested genotypes. Thus, under realistic conditions, the interaction of sex and natural selection makes the success of an allele due to its mixability in the current finite population indicative of its success as an interactant in future genetic combinations.

## Appendix

### Obtaining mixability ratios in the diploid case

In the diploid multi-locus model, for the $\left (\frac {n(n+1)}{2}\right)^{L}$ genotypes with *n* alleles and *L* loci, for each trial of the simulation, fitness values, $\widetilde {w}_{i_{1} j_{1}, i_{2} j_{2}, \ldots, i_{L} j_{L}}$ are drawn from the normal distribution $\mathcal {N}(E, \sigma)$ with average *E*=0.7 and standard deviation *σ*=0.15 and then truncated as described in the main text. If the fitness values of alleles $\hat {i}$ and $\hat {j}$ were adjusted as follows,

8 $$ {}w_{\hat{i}k, i_{2} j_{2}, \ldots, i_{L} j_{L}} = \widetilde{w}_{\hat{i}k, i_{2} j_{2}, \ldots, i_{L} j_{L}} \sqrt{\frac{d_{\hat{i}\hat{j}}\sum\limits_{k\neq\hat{i}; i_{2}, j_{2}, \ldots, i_{L}, j_{L}} \widetilde{w}_{\hat{j}k, i_{2} j_{2}, \ldots, i_{L} j_{L}}}{\sum\limits_{k\neq\hat{j}; i_{2}, j_{2}, \ldots, i_{L}, j_{L}} \widetilde{w}_{\hat{i}k, i_{2} j_{2}, \ldots, i_{L} j_{L}}}}  $$

and

9 $$ {}w_{\hat{j}k, i_{2} j_{2}, \ldots, i_{L} j_{L}} = \widetilde{w}_{\hat{j}k, i_{2} j_{2}, \ldots, i_{L} j_{L}} \sqrt{\frac{\sum\limits_{k\neq\hat{j}; i_{2}, j_{2}, \ldots, i_{L}, j_{L}} \widetilde{w}_{\hat{i}k, i_{2} j_{2}, \ldots, i_{L} j_{L}}}{d_{\hat{i}\hat{j}}\sum\limits_{k\neq\hat{i}; i_{2}, j_{2}, \ldots, i_{L}, j_{L}} \widetilde{w}_{\hat{j}k, i_{2} j_{2}, \ldots, i_{L} j_{L}}}},  $$

then the mixability ratio between $\hat {i}$ and $\hat {j}$ would have been nearly equal to $d_{\hat {i}\hat {j}}$ if the number of alleles *n* were sufficiently large. Due to computational restrictions, however, we run simulations for *n*=2, hence Eqs. (8) and (9) need to be changed to make the mixability ratio between $\hat {i}$ and $\hat {j}$ precisely equal to $d_{\hat {i}\hat {j}}$. In this case, for *L* loci, the fitness matrix has $\left (\frac {n(n+1)}{2}\right)^{L} = 3^{L}$ values. Notice that, for alleles $\hat {i}$ and $\hat {j}$ at the first locus, expression (8) increases the fitness of genotypes with $\hat {i}\hat {i}$ and $\hat {i}\hat {j}$ at the first locus at some given rate, and expression (9) decreases the fitness values of genotypes with $\hat {i}\hat {j}$ and $\hat {j}\hat {j}$ at the first locus at the same rate. Thus, the fitness values of genotypes with $\hat {i}\hat {j}$ at the first locus will be the same as in the beginning, and the ratio between the mixabilities of the pairs $\hat {i}\hat {i}$ and $\hat {j}\hat {j}$ (see [35] for the definition of mixability for k-tuples of interacting alleles) will be precisely $d_{\hat {i}\hat {j}}$. However, we need to get the ratio between the mixabilities of $\hat {i}\hat {i} + \hat {i}\hat {j}$ and $\hat {i}\hat {j} + \hat {j}\hat {j}$ to be equal to $d_{\hat {i}\hat {j}}$. Thus, to get the predefined mixability ratio $d_{\hat {i}\hat {j}}$, the fitness values of genotypes with $\hat {i}\hat {i}$ and $\hat {j}\hat {j}$ at the first locus should be adjusted differently.

Let *a*_*i*_ be the fitness values of genotypes with $\hat {i}\hat {i}, b_{i}$ be the fitness values of genotypes with $\hat {i}\hat {j}$, and *c*_*i*_ be the fitness values of genotypes with $\hat {j}\hat {j}$ at the first locus, and let $d_{\hat {i}\hat {j}}$ be a predefined mixability ratio. From Eqs. (8) and (9) it follows that

$$\frac{\sum a_{i}}{\sum c_{i}} = d_{\hat{i}\hat{j}}. $$

We would like to obtain

$$\frac{\sum a_{i} + \sum b_{i}}{\sum b_{i} + \sum c_{i}} = d_{\hat{i}\hat{j}}. $$

This is equivalent to

$$\frac{\sum a_{i}}{\sum c_{i}} = d_{\hat{i}\hat{j}} + \frac{\sum b_{i}}{\sum c_{i}} \left(d_{\hat{i}\hat{j}} - 1\right). $$

Since *b*_*i*_ and *c*_*i*_ are drawn from the same distribution $\mathcal {N}(0.7, 0.15)$, which implies that $\sum b_{i} = \sum c_{i}$, we get

$$\frac{\sum a_{i}}{\sum c_{i}} = 2d_{\hat{i}\hat{j}} - 1. $$

Hence, (8) and (9) are adjusted to

10 $$ \begin{aligned} w_{\hat{i}k, i_{2} j_{2}, \ldots, i_{L} j_{L}} = \widetilde{w}_{\hat{i}k, i_{2} j_{2}, \ldots, i_{L} j_{L}} \sqrt{\frac{\left(2d_{\hat{i}\hat{j}}-1\right)\sum\limits_{k\neq\hat{i}; i_{2}, j_{2}, \ldots, i_{L}, j_{L}} \widetilde{w}_{\hat{j}k, i_{2} j_{2}, \ldots, i_{L} j_{L}}}{\sum\limits_{k\neq\hat{j}; i_{2}, j_{2}, \ldots, i_{L}, j_{L}} \widetilde{w}_{\hat{i}k, i_{2} j_{2}, \ldots, i_{L} j_{L}}}} \end{aligned}  $$

and

11 $$ \begin{aligned} w_{\hat{j}k, i_{2} j_{2}, \ldots, i_{L} j_{L}} = \widetilde{w}_{\hat{j}k, i_{2} j_{2}, \ldots, i_{L} j_{L}} \sqrt{\frac{\sum\limits_{k\neq\hat{j}; i_{2}, j_{2}, \ldots, i_{L}, j_{L}} \widetilde{w}_{\hat{i}k, i_{2} j_{2}, \ldots, i_{L} j_{L}}}{\left(2d_{\hat{i}\hat{j}}-1\right)\sum\limits_{k\neq\hat{i}; i_{2}, j_{2}, \ldots, i_{L}, j_{L}} \widetilde{w}_{\hat{j}k, i_{2} j_{2}, \ldots, i_{L} j_{L}}}}. \end{aligned}  $$

### Derivation of expression (7)

As before, the theoretical probability of correct inference in the diploid multilocus model can be calculated as follows:

$$P(X<Y) = \iint \limits_{x< y} f_{X,Y}(x,y) dx dy, $$

where *X* and *Y* are random variables with joint probability density function *f*_*X*,*Y*_(*x*,*y*). Recall that *X* is drawn from the distribution of fitness values of genotypes that contain one allele of interest, $\hat {i}$, and *Y* is drawn from the distribution of fitness values of genotypes that contain another one, $\hat {j}$. The difficulty here is that these distributions are not independent because of the existence of a genotype that contains both alleles of interest.

Considering the case of two alleles per locus, let the genotypes at the first locus be $\hat {i}\hat {i}, \hat {j}\hat {j}$ and $\hat {i}\hat {j}$, and the fitness value distributions for each be the same as those of $\tilde {X}$ (for $\hat {i}\hat {i}$), $\tilde {Y}$ (for $\hat {j}\hat {j}$) and $\tilde {Z}$ (for $\hat {i}\hat {j}$). These three random variables are pairwise independent. We have, $X = \tilde {X} + \tilde {Z}$ and $Y = \tilde {Y} + \tilde {Z}$. Therefore,

$$\begin{array}{*{20}l} P(X<Y) &= P\left(x< y | x \in X, y \in Y\right)\\ &= P\left(\tilde{x} + \tilde{z} < \tilde{y} + \tilde{z} | \tilde{x} \in \tilde{X}, \tilde{y} \in \tilde{Y}, \tilde{z} \in \tilde{Z}\right) \\ &= P\left(\tilde{x} < \tilde{y} | \tilde{x} \in \tilde{X}, \tilde{y} \in \tilde{Y}\right) = P\left(\tilde{X} < \tilde{Y}\right), \end{array} $$

where $\tilde {X}$ and $\tilde {Y}$ are independent.

### Multilocus binary models

#### Multilocus haploid binary model

One of the causes of random deviations from “correct evaluation” of mixabilities in the multilocus haploid model was the probabilistic nature of survival. Here, we carry out a similar simulation with binary fitness values, such that the values of each genotype can be either 0 or 1. Now the mixability of an allele is calculated by dividing the number of genotypes of fitness 1 that carry this allele by the total number of genotypes that carry this allele. $d_{\hat {i}\hat {j}}$ is equal to the ratio of these fractions for the two alleles of interest.

The starting population consists of concrete genotypes as in the main haploid model, but here we must ensure that all parents that survived to replicate have fitness 1 (i.e., there is no genotype with fitness 0 in the starting population), and that the mixabilities of the two alleles of interest ($\hat {i}$ and $\hat {j}$) at the first locus closely approximate some predefined values. One way to do so, is to place zeros and ones in the fitness matrix at the rate that would lead to the mixability ratio, $d_{\hat {i}\hat {j}}$, chosen for the given simulation.

Note that, if the number of loci *L* is small relative to the population size and the starting allele frequencies are equal, then the set of parents may include too many possible genotypes, leaving no room for enough zero values in the fitness matrix. Therefore, we can run the simulation of discrete fitness values for haploid multilocus model for sufficiently large values of *L*, depending on the population size *N* and the number of alleles per locus *n*.

Appendix Fig. 15 shows the result of such a simulation for a population size of 2000 haploids, $d_{\hat {i}\hat {j}}$ values ranging from approximately 1.01 to 1.11, *n*=2 alleles per locus and 100 independent trials for each parameter combination. For the selected population size and number of alleles, the number of loci *L* is *L*≥11. Results are analogous to those of Fig. 1: the number of tested genotypes is similar across all panels, *P* is increasing with $d_{\hat {i}\hat {j}}$ but not with *L*, and the confidence intervals are similar. For example, for 16 loci, the confidence interval of *P* increases from (0.47,0.67) for $d_{\hat {i}\hat {j}} = 1.0112$ to (0.95,1) for $d_{\hat {i}\hat {j}} = 1.1111$ in Appendix Fig. 15 and from (0.49,0.67) for $d_{\hat {i}\hat {j}} = 1.0112$ to (0.96,1) for $d_{\hat {i}\hat {j}} = 1.1111$ in Fig. 1.

Bar plots for distributions of fitness values for the two alleles of interest in the case of 18 loci and three values of $d_{\hat {i}\hat {j}}$: 1.0112, 1.0227 and 1.0345 are shown in Appendix Fig. 16. We see that the difference between these distributions is increasing with the mixability ratio. Also, the mixabilities of given alleles are precisely equal to the predefined values (0.9 for the more mixable allele and from 0.89 to 0.87 for the less mixable one).

As in the main multilocus haploid model, we have examined not only hermaphrodites capable of selfing but also the case of two mating types. Results are very similar to those of Appendix Fig. 15 (not shown). Additionally, we have performed the sampling of parents and of alleles within parents without replacement to observe the “pure” effect of random sampling by sex free of the effects of drift (Appendix Fig. 17). Results are much stronger than those in Appendix Fig. 15 and are very similar to those in Fig. 2.

#### Sex vs. asex in the haploid binary model

Here we examine the mixability prediction for a range of recombination values and thus are able to compare asex (*r*=0) to sex (*r*=0.5 in the free recombination case), starting at linkage disequilibrium, where the initial population consists of distinct clones, with discrete fitness values, 0 and 1. Because of this, offspring in the asexual case always survive. Therefore all alleles of the population created in the asexual case during the simulation have equal frequencies, there is no difference between alleles $\hat {i}$ and $\hat {j}$, and the fraction of trials, *P*, in which the allele that is more mixable across all possible genotypes increases in frequency more than the allele that is less mixable across all possible genotypes, is precisely 0.5.

In contrast, in the sexual population, any shuffling of alleles produces more combinations than existed originally (and the number of tested genotypes increases with the recombination rate). Thus *P* increases substantially even for small values of *r* (Appendix Fig. 18).

**Fig. 7:**
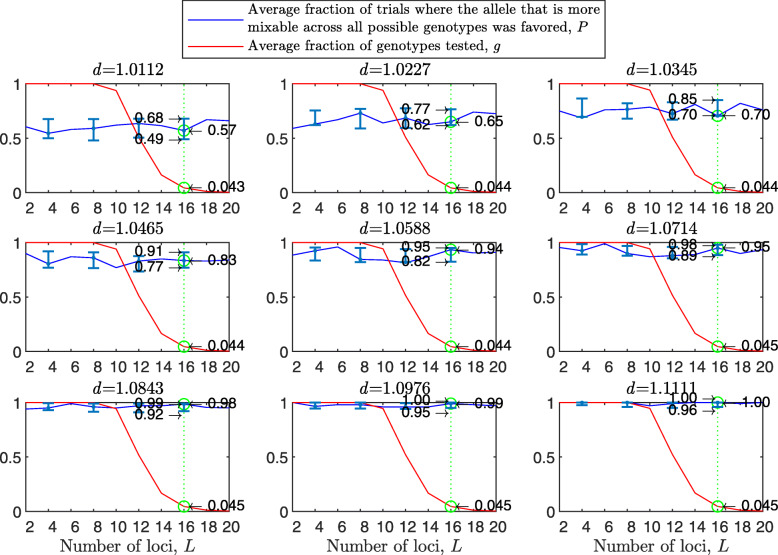
Random sampling in a multi-locus haploid model with two mating types. The simulation conditions are as described in Fig. 1, except that now the parents are divided into two mating types, so that mating can occur only between type 1 and type 2 individuals

#### Multilocus diploid binary model

For the multilocus diploid binary model with *L* loci and *n* alleles per locus, each trial run of the simulation is similar to the haploid binary model. In the beginning, the starting population of random parents is created in such a way that all alleles in this population have equal frequencies. Then a fitness matrix with binary values is created. This step has some conceptual differences from the haploid binary model. We have $\left (\frac {n(n+1)}{2}\right)^{L}$ genotypes as in the general diploid case. However, at the stage of filling the fitness matrix with zeros and ones at some rate, we should take into account that for two alleles of interest $\hat {i}$ and $\hat {j}$ at the first locus, in the diploid model we have genotypes that contain both these alleles.

As in the haploid binary model, the simulation can be run if the number of loci, *L*, is relatively large. Appendix Fig. 19 shows the results of such a simulation with population size *N*=2000, number of loci *L* from 7 to 15 and two alleles per locus in a manner analogous to Fig. 5. The results are the same as in the multilocus diploid model, i.e., stronger than any of the haploid models. Examine the green lines positioned at 16 loci for the haploid model in Appendix Fig. 15 and at 12 loci for the diploid model in Appendix Fig. 19. The 95% confidence interval increases from (0.47,0.67) in the haploid and (0.56,0.73) in diploid models in the top-left panel to (0.82,0.94) in the haploid and (0.92,0.99) in diploid models in the central panel to (0.95,1.00) in the haploid and (0.99,1.00) in diploid models in the bottom-right panel.

#### Sex vs. asex in the diploid binary model

We compared sex and asex for the binary fitness matrix in the diploid case. The starting population consists of two clones, for example, (0_1_0_1_;0_2_0_2_;…;0_*L*_0_*L*_) and (1_1_1_1_;1_2_1_2_;…;1_*L*_1_*L*_), where 0_*l*_ and 1_*l*_,1≤*l*≤*L* are two alleles, and two generations are computed and tracked.

The results of this simulation are presented in Appendix Fig. 20 in a manner analogous to Figs. 18 and 6. In comparison to the haploid case (see Appendix Fig. 18), the confidence interval is narrower and higher. Take for example a green line drawn in each panel for a recombination rate of 0.3. For the small mixability ratio *d*=1.0112, the difference between the haploid and diploid cases is only in the size of 95% confidence interval: while for the haploid case it is (0.47,0.66), for the diploid case it is (0.49,0.64). This difference becomes stronger in the central panel for *d*=1.0588: from (0.67,0.84) for the haploid case to (0.77,0.90) for the diploid case. Finally, in the bottom-right panel, the confidence intervals for the haploid and diploid binary models are different: (0.80,0.94) for the haploid vs. (0.93,0.99) for the diploid. The reason for this difference lies in Eqs. (5) and (6) as explained earlier.

## Appendix figures

**Fig. 8:**
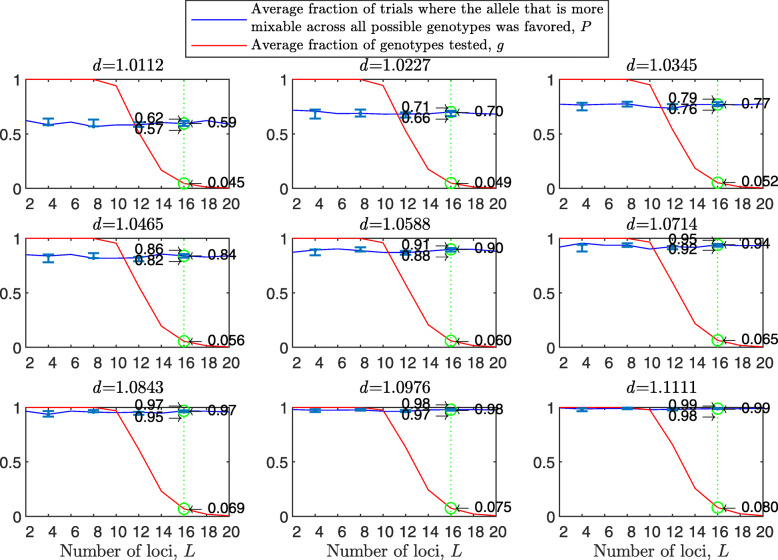
Random sampling in a multi-locus haploid model when all loci are tracked simultaneously. In each panel, results are shown for a population size of 2000, a varying number of loci from 2 to 20, and 2 alleles per locus, based on 100 independent trials. Bars for the 4, 8, 12 and 16 alleles represent 95% confidence interval for *P* based on 80 values, each of which was obtained based on 100 independent trials. *P* now refers to all loci rather than one (see main text). In comparison to the analysis of the first locus case in Fig. 1, *L* times more transformations are applied here to the fitness matrix. This leads to a decrease in both its variance (the reason for the thinner confidence interval of *P*) and average (the reason for the increase of *g* because more genotypes need to be created to obtain *N* surviving individuals). To facilitate comparison, the green line highlights the results for 16 loci case in Figs. 1 and 8

**Fig. 9:**
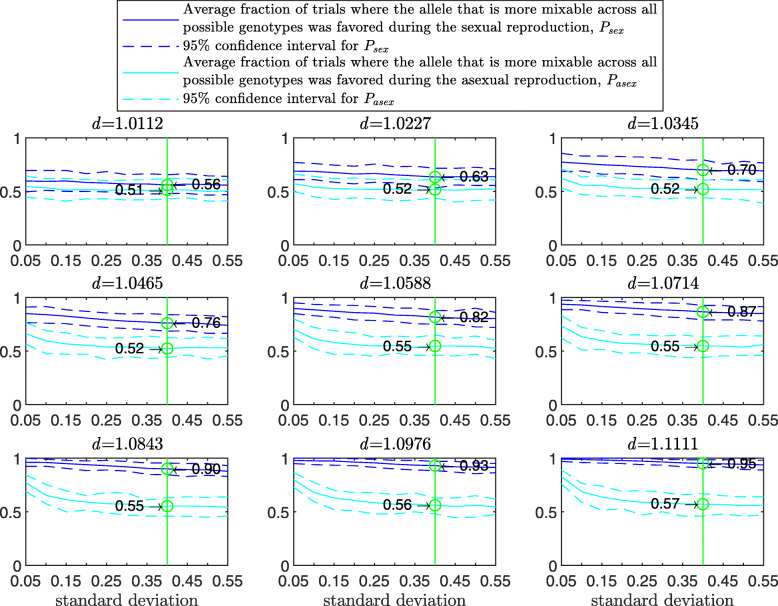
Comparison of sampling by sex and asex in the multi-locus haploid model for different standard deviations of the initial fitness values distribution. The simulation conditions are as described in Fig. 4, except that now the population size is fixed (*N*=2000) and the standard deviation of the fitness distribution varies. This figure shows that as the standard deviation increases, *P* decreases rapidly to almost 0.5 in the asexual population, while in the sexual population it decreases far more slowly in an apparently linear fashion

**Fig. 10:**
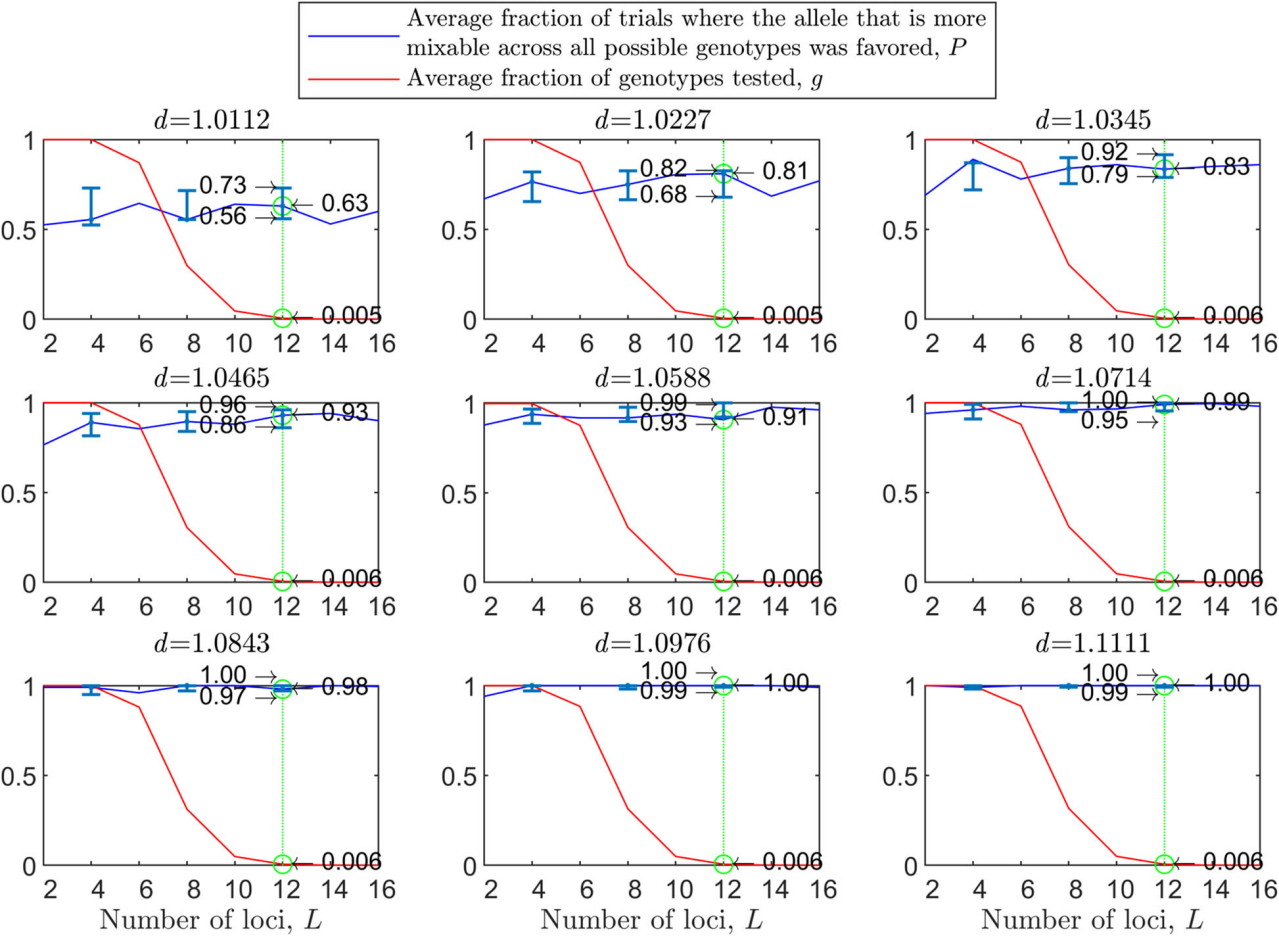
Random sampling in a multi-locus diploid model with two mating types. The simulation conditions are as described in Fig. 5, except that now the parents are divided into two mating types, so that mating can occur only between type 1 and type 2 individuals

**Fig. 11:**
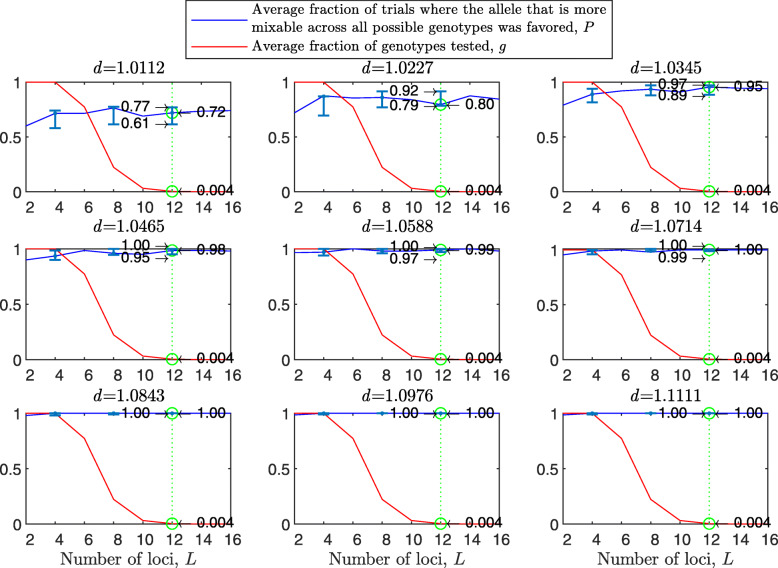
Random sampling in the multi-locus diploid model without random genetic drift. Random sampling in the multi-locus diploid model with fitness values from the normal distribution $\mathcal {N}(0.7, \, 0.15)$, two mating types and without replacement of parents and alleles. The simulation conditions are as described in Fig. 5, except that now the parents are divided into two mating types, each parent participates in exactly two reproductive events, and each allele in each parent is transmitted exactly once. The difference between the present figure and Fig. 5 shows the importance of drift due to the sampling of parents and of alleles with replacement

**Fig. 12:**
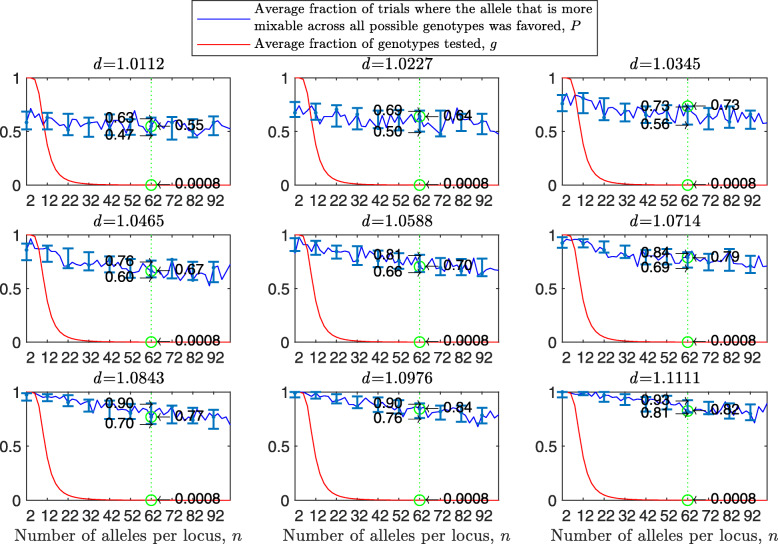
Random sampling in a diploid model with two loci and a different number of alleles. The simulation conditions are as described in Fig. 5, except that now the number of loci is fixed (*L*=2) and the number of alleles per locus, *n*, varies. This figure shows that as *n* increases, *P* decreases

**Fig. 13:**
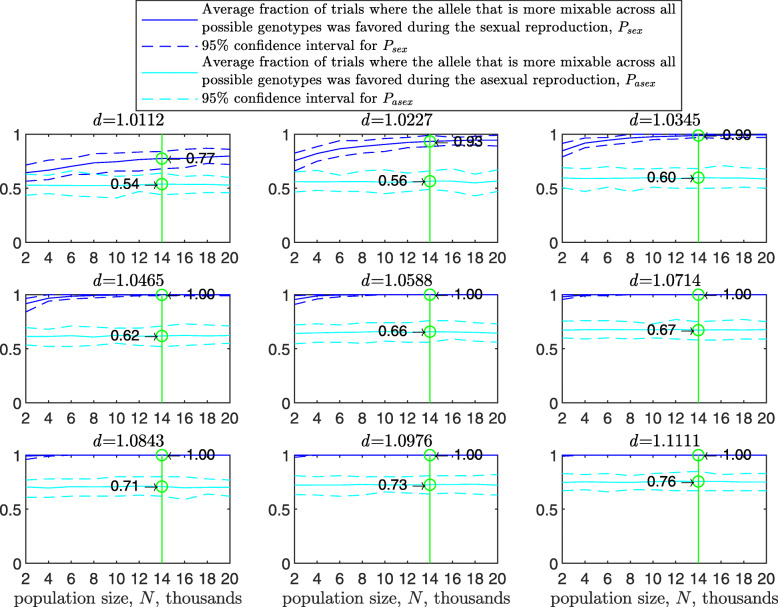
Comparison of sampling made by sex and by asex in the multi-locus diploid model for different population sizes. The simulation conditions are as described in Fig. 6, except that now the population size varies on the *x*-axes and only two recombination rate values are used, *r*=0 (asex, cyan solid line; 95% C.I. cyan dashed lines) and *r*=0.5 (sex, blue solid line; 95% C.I. blue dashed lines). This figure shows that *P* is much higher in the sexual than in the asexual population, and that as the population size is increased, *P* increases further in the sexual but not in the asexual population

**Fig. 14:**
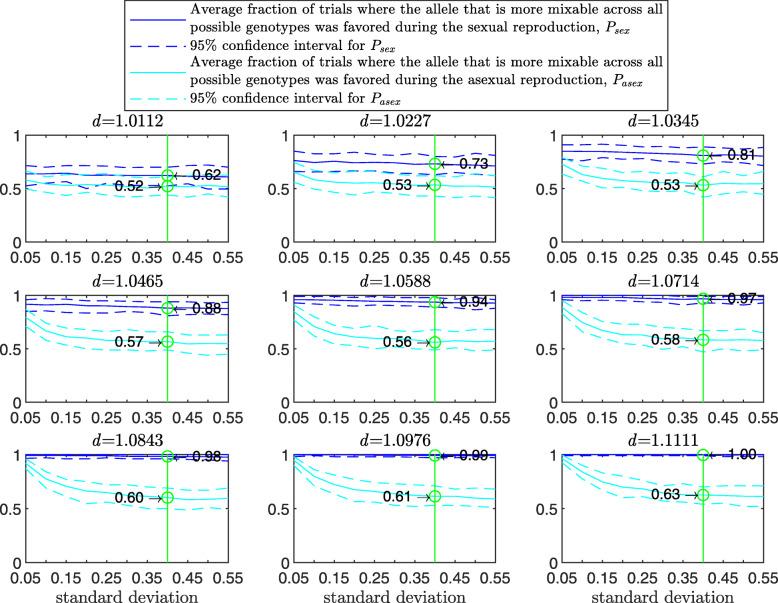
Comparison of sampling made by sex and by asex in the multi-locus diploid model for different standard deviations of the initial fitness distribution. The simulation conditions are as described in Appendix Fig. 13, except that now the population size is fixed (*N*=2000) and the standard deviation of the fitness values, *σ*, varies. This figure shows that as *σ* increases, *P* decreases rapidly in the asexual population to 0.52−0.63, while in the sexual population it decreases slowly in an apparently linear fashion. The maximum difference between *P* in the sex and asex cases is for *σ* of approximately 0.3−0.4

**Fig. 15:**
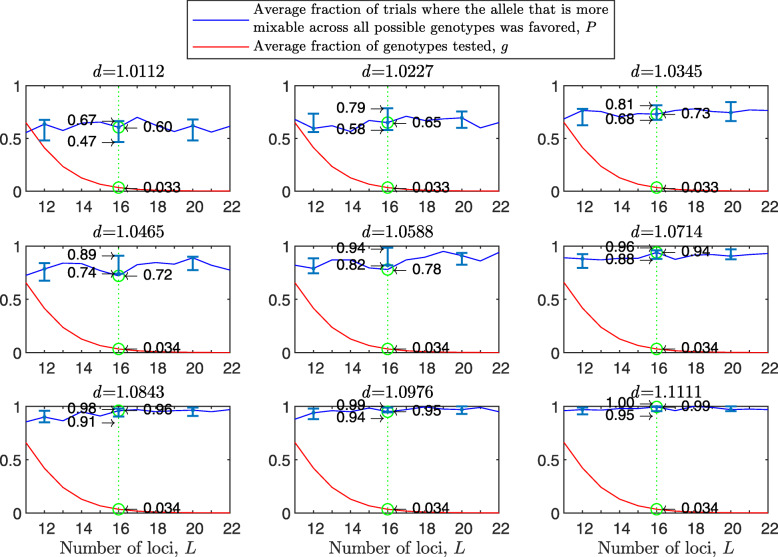
Random sampling in a multi-locus haploid model with binary fitness values. The fraction of genotypes of fitness 1 out of all possible genotypes for the more mixable allele is 0.9 for each panel, whereas the fraction of genotypes of fitness 1 out of all possible genotypes for the less mixable allele decreases from 0.89 to 0.81, producing a range of *d* values (the ratio between the fractions of genotypes of fitness 1) from 1.0112 to 1.1111

**Fig. 16:**
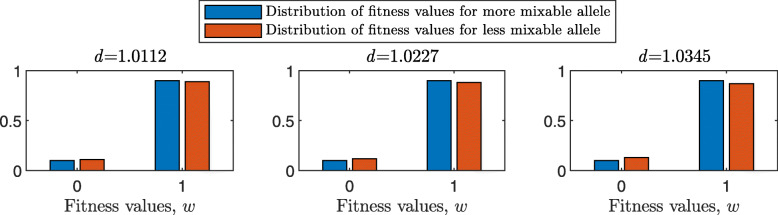
Distribution of fitness values in the haploid multi-locus model with binary fitness values. Each pair of bars shows the fraction of zeros or ones in the fitness matrix for one of the alleles of interest, for a population size of 2000, 18 loci and 2 alleles per locus. The fraction of genotypes of fitness 1 for the more mixable allele is 0.9, whereas the fraction of genotypes of fitness 1 for the less mixable allele decreases from 0.89 to 0.87, producing 3 *d* values. The left bar-chart shows two pairs of bars for alleles whose fractions of genotypes with fitness 1 are equal to 0.9 and 0.89, respectively, producing a mixability ratio $d = \frac {0.9}{0.89} \approx 1.0112$. The right bar-chart represents two pairs of bars for alleles whose fractions of genotypes with fitness 1 are equal to 0.9 and 0.87, respectively, producing a mixability ratio $d = \frac {0.9}{0.87} \approx 1.0345$. The first pair of bars in each panel shows the fraction of zero values in the fitness matrix and the second pair shows the fraction of ones. Note that the difference between the more mixable allele and the less mixable allele increases with *d*

**Fig. 17:**
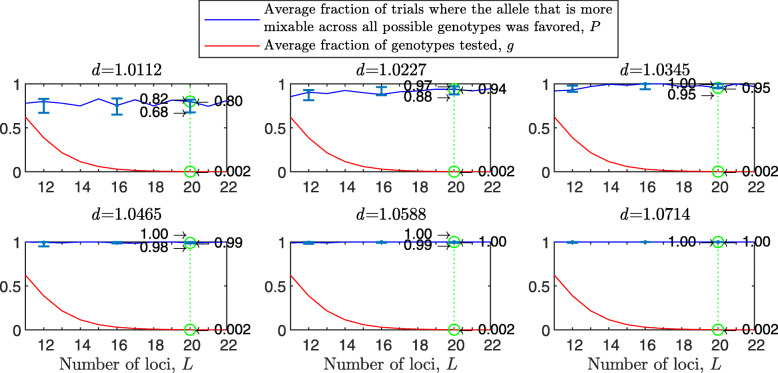
Random sampling in the multi-locus haploid model with binary fitness values, two mating types and without replacement of parents and alleles. The simulation conditions are as described in Appendix Fig. 15, except that now the fitness values are binary and parents are divided into two mating types, so that mating can occur only between type 1 and type 2 individuals. Each parent participates in exactly one reproductive event, which creates two offspring, such that each allele in each parent is transmitted exactly once. The difference between the present figure and Appendix Fig. 15 shows the importance of drift due to the sampling of parents and of alleles with replacement

**Fig. 18:**
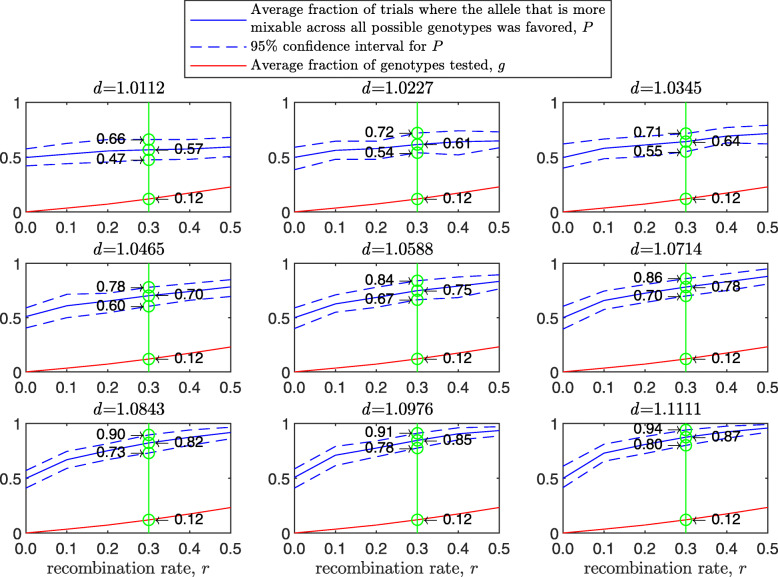
Comparison of sampling made by sex and by asex in a multi-locus haploid model with binary fitness values. The simulation process is similar to Fig. 3, except that now the fitness values are binary

**Fig. 19:**
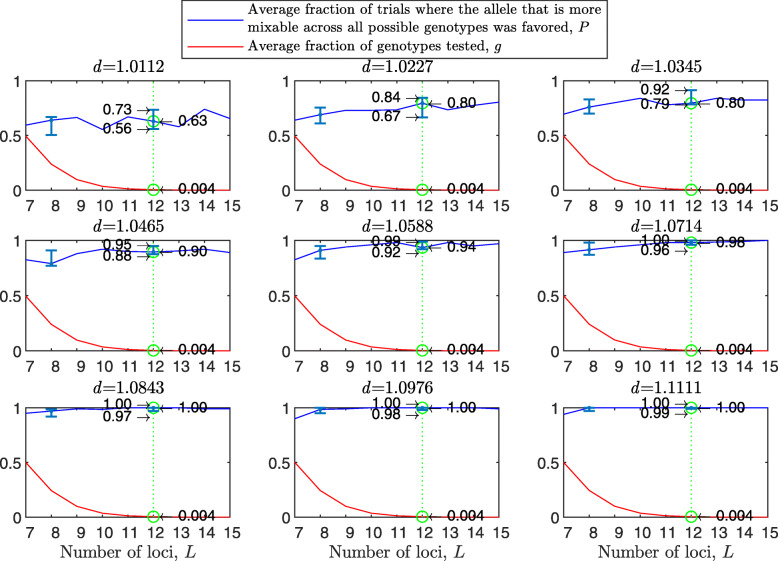
Random sampling in a multi-locus diploid model with binary fitness values. The results are produced in a manner analogous to Appendix Fig. 15, the difference being that this model is diploid and the number of loci ranges from 7 to 15. The results in this model are much stronger than in the haploid case

**Fig. 20:**
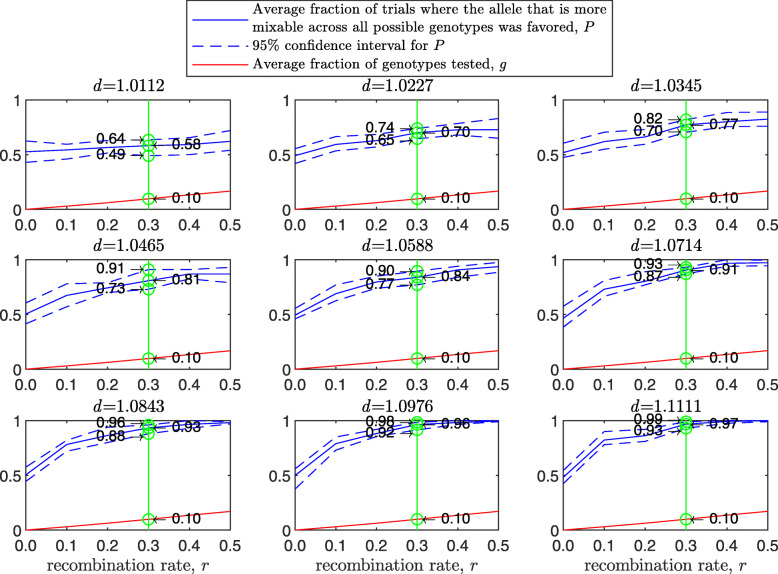
Comparison of sampling made by sex and by asex in the multi-locus diploid model with binary fitness values. In each panel, results are shown for a population size of 2000, 8 loci and for 2 alleles per locus. The simulation conditions are as described in Fig. 6, except that now fitness values are either 0 or 1

## Data Availability

All data generated or analysed during this study are included in this published article.
